# Theoretical Mapping of Suicidal Risk Factors During the COVID-19 Pandemic: A Mini-Review

**DOI:** 10.3389/fpsyt.2020.589614

**Published:** 2021-01-22

**Authors:** Saurabh Raj, Debasruti Ghosh, Tushar Singh, Sunil K. Verma, Yogesh K. Arya

**Affiliations:** ^1^Department of Psychology, Ramdayalu Singh College (Babasaheb Bhimrao Ambedkar Bihar University), Muzaffarpur, India; ^2^Department of Psychology, Mahant Darshan Das Mahila College (Babasaheb Bhimrao Ambedkar Bihar University), Muzaffarpur, India; ^3^Department of Psychology, Faculty of Social Sciences, Banaras Hindu University, Varanasi, India; ^4^Department of Applied Psychology, Vivekananda College, University of Delhi, New Delhi, India; ^5^Department of Psychology, Faculty of Social Sciences, Banaras Hindu University, Varanasi, India

**Keywords:** burdensomeness, thwarted belongingness, cognitive distortions, COVID-19, suicide, diathesis

## Abstract

Suicide prevention in times of COVID-19 pandemic has become more challenging than ever due to unusual circumstances. The common risk factors identified with regard to suicidal behavior are fear of COVID-19, economic instability, poor access to healthcare facilities, pre-existing psychiatric disorders, and social disconnect. The studies done so far have reported either case studies or have made an effort to understand the risk factors. An understanding of the underlying causal pattern from existing theories, behind these risks, will enable adopting appropriate prevention mechanisms. Hence, this review examines evidence related to risk factors of suicides that occurred during COVID 19 and discusses it in the light of three major theoretical approaches: interpersonal model, stress diathesis model, and cognitive model. The insights obtained from the three viewpoints reveal that perceived burdensomeness, thwarted belongingness, stress sensitivity, cognitive errors such as magnification, catastrophic thinking, arbitrary inference, and mind-reading are likely reasons behind these risk factors for suicide. It is suggested that awareness regarding COVID-19 stressors, use of community-based approaches like gatekeeper training, and brief online psychotherapy by using techniques of mindfulness, interpersonal psychotherapy, and cognitive behavior therapy can be useful in reducing suicide risk during COVID-19.

## Introduction

The novel coronavirus pandemic is an unimaginable life event that has impacted each and every individual in different ways. The direct effects of this disease are related to health but indirectly, the containment measures adopted to counter the contagious infection have created a plethora of socioeconomic and psychological repercussions (1, 2). People with psychiatric conditions, especially those who require institutionalized care and in-person psychotherapy sessions, have been adversely affected (3). In absence of proper medical care, individuals with psychiatric disorders may witness a change in activities of daily living, sleep-wake patterns, social rhythms, and heightened emotional reactions (4). Besides, indirectly this pandemic is a precipitating factor for people who have developed mood problems due to an overwhelming crisis. For example, Hawryluck et al. conducted a study during the SARS epidemic and demonstrated that the quarantine period could be a triggering factor for psychological distress and a longer period of quarantine was associated with depression and post-traumatic stress disorder (5).

It has been observed that suicide rates generally increase after disasters. A higher rate of completed suicide was observed in victims of the 1999 Taiwan Earthquake (6). A study during the SARS outbreak in Taipei reported an increase in suicides after strict quarantine measures were imposed (7). Similar increasing trends in the number of suicides was seen in older adults who had a chronic illness or functional impairment during the SARS outbreak in Hong Kong in 2003 (8, 9). Suicide and self-harm behaviors have become pressing concerns during this COVID-19 pandemic as well. Some of the most potent risk factors for suicides identified by researchers during the COVID-19 pandemic are social isolation, economic downturn due to lockdown, increase in anxiety and stress in healthcare professionals (10), interpersonal violence (11), and stigma and discrimination (12). Thakur and Jain (2020) reported two suicidal cases from India, one because of wrongly interpreting the infection and the other one because of social isolation (10). An analysis of 72 suicide cases during the COVID-19 period in India reported that fear of getting infected with the virus was the most prominent reason for suicide incidence (13). These case studies and cross-sectional evidence give an idea about COVID-19 stressors and their relation to suicidal behavior. A recent systematic review on self-harm and suicide rates presented an analysis of modeling-based studies that estimated the effect of the pandemic on rise of suicidal cases which ranged from 1 to 145% (14).

However, the above-mentioned studies do not provide a clear understanding of the underlying causal patterns behind the emergence of risks. These risk factors have their origins in various cognitive errors, dysfunctional thinking patterns, traits vulnerability, and interpersonal attributes (15, 16). An understanding of these patterns can be important in giving direction to crisis intervention strategies to deal with suicidal behavior in such emergencies. Hence, the present review aims to discuss available literature that has identified the risk factors for suicides during COVID-19 and present them in the light of existing theoretical models. The findings from the relevant literature have been explained in the background of the interpersonal model of suicide, the stress diathesis model, and the cognitive model. Suicide risk factors have been explained by several theoretical viewpoints, but this review focuses on these three paradigms as they offer a broader understanding of the nature of risk factors related to suicides in this unprecedented pandemic. An implication from the theory helps in understanding the severity of risk factors and tracing the genesis of such a maladaptive pattern. In the review, we have used data from secondary sources and recent reported case studies, to highlight the increase in mood-related problems and subsequent increase in suicidal ideation and attempts. The paper also summarizes some of the psychological interventions that could help in dealing with suicide risk, some of which have been drawn from model-based approaches that have been used in this review.

## Explanation of Suicide Risks Through the Interpersonal Model

The two major components of the interpersonal theory of suicide, i.e., thwarted belongingness and perceived burdensomeness have been attributed as reasons for suicidal behavior (17). Thwarted belongingness represents a psychological state in which the need for social connectedness and the need for belongingness are not adequately met (18). Social isolation, loneliness, and lack of social support are indicators that belongingness needs are unfulfilled. Many studies have linked loneliness as a risk factor for suicidal ideation and suicide attempts (19, 20). The current social exchange situation created by the COVID-19 pandemic requires the practice of social distancing, which may inadvertently lead to feelings of loneliness and isolation (21). Perceived burdensomeness is a state where an individual feels that he or she is a burden on others and that others will be better without his or her existence. Individuals with chronic illness, unemployment, and family discord can develop this sense of burdensomeness, which in turn can act as a trigger to suicidal behavior.

**Figure 1:**
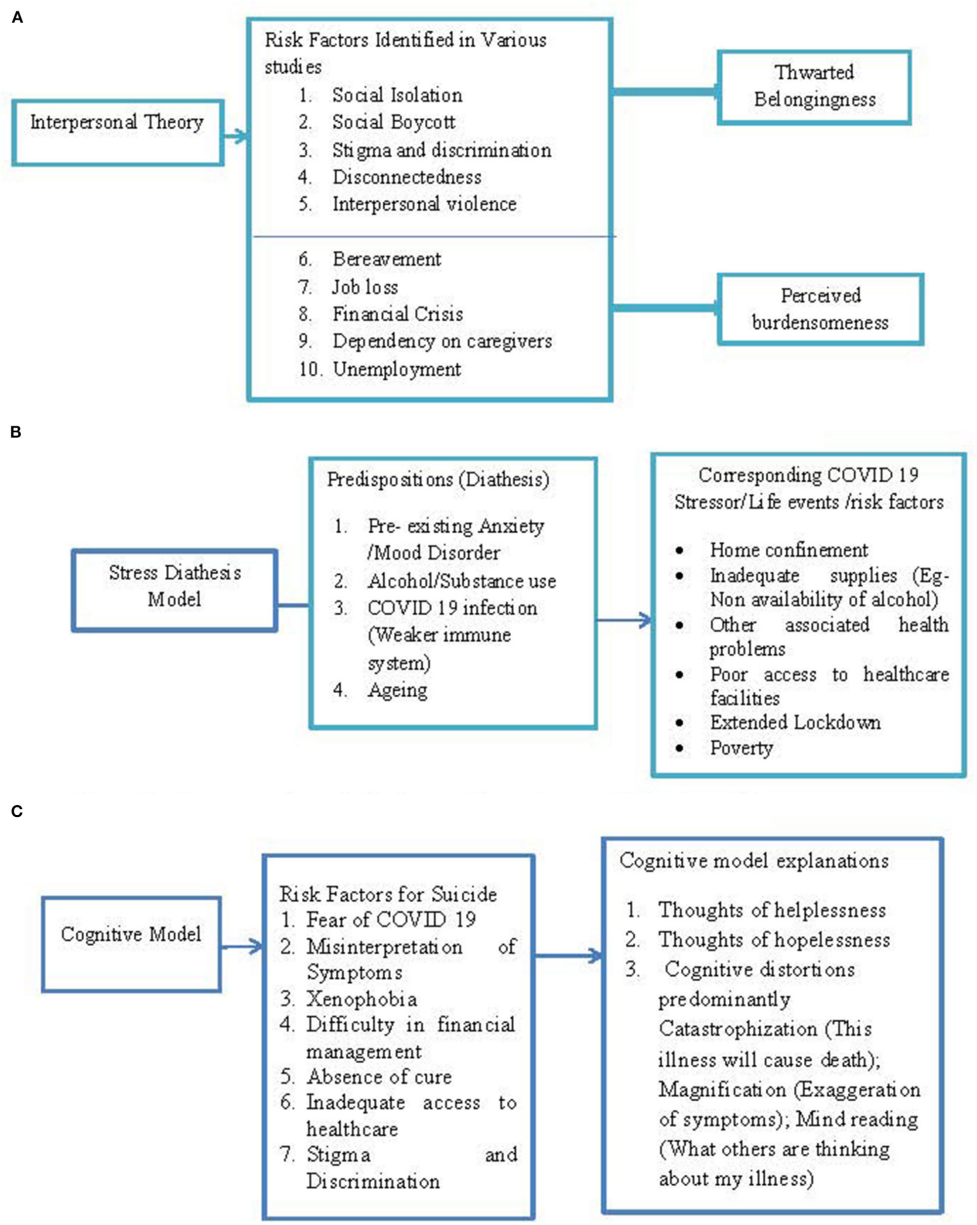
**(A)** Representation of risk factors through interpersonal model. **(B)** Representation of risk factors through stress diathesis model. **(C)** Representation of risk factors through cognitive model.

A recent study reported that the stay-at-home order, due to spread of COVID-19, was indirectly associated with suicide risk because of greater thwarted belongingness (22). The study explained that social disconnection and the absence of meaningful relationships were responsible for the association between stay-at-home orders and suicide risk. Research on older adults has also found that people of this age group are at higher suicide risk and may develop anxiety and depression due to this physical distancing, social disconnectedness, and perceived isolation (23). The risk factors identified in the studies can pose a threat to the belongingness needs of the individual and make them vulnerable to stress and suicide (see Figure 1A). A few reports claim that self-harm or suicidal tendencies in people of old age have increased, particularly in those who are dependent on their caregivers or family for their needs during this pandemic (24). A possibility of a perception of burdensomeness due to dependency and loss of productivity can reinforce such behaviors.

Studies based on rural-urban settings have highlighted that people living in rural areas are at increased suicidal risk as they experience more social isolation (11). The reason given for such an assumption is that generally, rural areas are less densely populated; hence, social support can be more difficult to obtain during acute suicidal crises. In such circumstances, the social disconnect can give rise to thwarted belongingness resulting in suicidal tendencies. Reports on attempted or completed suicides from media that have been cited in case studies have mentioned burden due to unemployment and job loss (25, 26); social boycott, discrimination, bereavement, and loss of loved ones (13) are also risk factors for suicidal behavior. These suicide factors can be traced through interpersonal theory constructs of burdensomeness and thwarted belongingness. A person who has lost a job recently or is unemployed might think that he is a burden on his family members. Social discrimination and feelings of stigma can be interpreted as signs of non-acceptance from societal members leading to a thwarted belongingness state.

Among other vulnerabilities traumatic experiences due to interpersonal violence, such as intimate partner violence, have shown an upward trend in COVID-19 (27). There have been instances of suicide cases due to these factors also. It has been speculated that substance use, economic volatility, and poverty have perpetuated reactions in men in the form of aggression against women (28). The trauma experienced by women due to interpersonal violence challenges the belongingness needs and is associated with dysphoric arousal and suicidal risk (29).

## Explanation of Suicide Risks Through the Stress Diathesis Model

The stress diathesis model views suicide as a resultant action of a predisposition (diathesis) and an event (stressor) that triggers stress reactions in an individual and increase the susceptibility to attempt suicide (30). In general, the diathesis depends on certain traits in the psychological or clinical profile of the individual. Recent research has linked COVID-19 stressors such as self-isolation, being socially cut off, and economic uncertainties with higher traumatic stress in people with preexisting mood disorders (31). The study also mentions that people with anxiety-related disorders reported a greater level of distress due to fear of contamination, xenophobia, and perception of danger.

Stress-related disorders, sleep disorders, and mood disorders are strongly associated with suicidal behavior (32). A recent cross-sectional study from Colombia in the context of COVID-19 concluded that depressive symptoms due to confinement and insomnia are associated with higher perceived stress and higher suicide risk (33). Case studies based on newspaper reports have identified reasons where substance users committed suicide due to the non-availability of alcohol as shops were closed by abrupt lockdown (13). Thus, a preexisting psychiatric condition in combination with an active life event like COVID-19 and its related stressors further aggravates the stress and increases vulnerability to suicide. Instances from older adults committing suicide revealed that they had a preexisting medical condition and feared that they would be infected by COVID-19 (13). Age-related illnesses and chronic conditions such as diabetes and hypertension increase vulnerability (diathesis) and, when accompanied by fear of COVID-19 (stressor), lead to distress and suicidal behavior.

Survivors of COVID-19 infections are also at an increased risk for suicide (34). A recent study has shown that about 25% of COVID-19 patients experience neurological problems, too (35). It has been observed that COVID-19 patients are reporting dizziness, headache, seizures, ischemic stroke, and other neurological problems (34, 36). The idea of COVID survivors' increased risk for suicide is supported by the findings of a previous study, which reports that neurological disorders such as ischemic stroke and headaches are associated with increased propensity to suicide (37). This evidence points to links between COVID-19 infection leading to other neurological problems and suggests that it might be the cause of stress reactions. On the other hand, other evidence has speculated that COVID-19 causes mood symptoms. It has been hypothesized that immune system responses to SARS CoV-2 may induce mood symptoms and suicidal ideation in some subpopulations (38). These speculated links to suicide provide an alternate explanation whereby the weakened neurological and immunological response aggravates susceptibility to stressors and induces mood conditions and suicidal tendencies (see Figure 1B).

## Explanation of Suicide Risk Through the Cognitive Model

The cognitive approach has provided compelling evidence about the development of depressive cognition and suicide. The nature of COVID-19 is such that its related stressors are uncontrollable, which can make individuals apprehensive and helpless. Lack of a cure, poor access to medical facilities, and uncertainties regarding the end of pandemic can prompt feelings of hopelessness. These feelings are backed by automatic negative thoughts, which could be a possible explanation for cases of suicide reported due to financial instability, employment status, postponement of exams, and inadequate supplies during lockdown (26, 39). Additionally, several cognitive distortions can perpetrate negative thinking patterns and form the basis of suicidal thoughts/behavior (15). The case studies dealing with suicide attempts due to circumstances involving COVID-19 provide the impression that dysfunctional thought patterns could be a possible cause of negative emotions and suicidal attempts. There are cases where people thought they were COVID-19 positive and experiencing symptoms of the disease, because of a misinterpretation of their flu symptoms/illnesses (13, 39, 40). These misinterpretations can be understood in the context of “magnification” and “arbitrary inference” cognitive distortions. The symptoms of one illness that are similar to those of COVID-19 can lead to the tendency to overgeneralize, and people may possibly overlook other evidence. These distortions might lead to negative thoughts related to dying (see Figure 1C).

Another prominent cognitive distortion for suicidal behavior seems to be “catastrophic thinking.” Suicide attempts due to fear of contracting COVID-19, postponement of exams, and being COVID-19-positive could be related to this cognitive distortion. Sahoo et al. reported two cases, the first of which was of a person who shot himself while he was in self-isolation. He developed mood symptoms after coming in contact with a COVID-19-positive person and isolated himself. As per the report he was preoccupied by thoughts of dying from COVID-19 and therefore committed suicide (41). The same study reported an attempted suicide by an individual who was asked to take a photograph of a foreign couple, while he was on his morning walk. Later, he was in panic when he came to know about the transmission mode, death rates, and links to foreign travel via media and news channels. He was in a state of social withdrawal and assumed that he was going to die and therefore attempted suicide (41). These individuals imagined the worst outcomes demonstrating catastrophic thinking.

Suicidal deaths due to the stigma related to COVID-19, xenophobia, and social discrimination can be understood through the lens of the “mind reading” cognitive distortion. This distortion is described as a tendency to overemphasize and misjudge others' perception of them. In two such noticeable cases, it was found that the reason for suicide was the perception of discrimination and xenophobia. These individuals worked in a different place, and due to lockdown they returned to their villages. They believed that the villagers were thinking about them negatively and discriminating against them, and so they committed suicide (32, 42).

## Discussion and Recommendations

This review is an attempt to establish the theoretical links behind risk factors for suicides during COVID-19. The risk factors identified have been discussed in consideration of three major theoretical models, i.e., the interpersonal model, the stress diathesis model, and the cognitive model. Interestingly, all the models offer an elaborative explanation of the risk factors such as isolation, age-related suicide, preexisting mood disorders, chronic illness, poverty, unemployment, and fear of COVID-19. A recent study in the context of COVID-19 determining youth's susceptibility for high risk for psychosis emphasizes stress sensitivity, diathesis-stress model, and cognitive biases as the potential factors (43). Thwarted belongingness and perceived burdensomeness have been previously linked to suicide risk during the Red River flood disaster (44). Dysfunctional thought patterns and cognitive errors of catastrophic thinking, magnification, arbitrary inference, and mind-reading have been reported in cases of suicide after stressful life events (15, 45). The insights generated through the explanations in this integrated model can be very useful in chalking out crisis interventions during such emergencies.

Suicidal behavior in itself is an emergency condition that needs efficient crisis intervention strategies. Many countries have responded to this crisis by setting up psychosocial support and suicide helplines to respond to an immediate crisis. Apart from these support systems, efforts are being made to raise awareness regarding the symptoms of COVID-19 and take care of mental health (3). However, the consequences of COVID-19 stressors are appalling and highly uncertain. Special populations, such as older adults, people with preexisting psychiatric illness, and people with mood symptoms, may need further support. The current pandemic poses an additional challenge to the practice of psychological interventions through in-person settings. Hence, digital modalities such as web-based counseling, telepsychotherapy, and teleconsultations can be used as alternatives. Although web-based modalities are not free from limitations, such as the absence of non-verbal cues, problems related to inhibition, and temporal fluidity (46). But in times of physical distancing, they can be used to address crisis-related issues. Healthcare professionals, especially psychologists and social workers, can train caregivers, nurses, and staff, as well as family members, to learn and practice suicide assessment and brief interventions online (47, 48).

The gatekeeper training approach can be very useful in identifying people at risk for suicide (49). The training involves teaching a certain group of people to identify, assess, and refer people who are at increased suicide risk. These people can be primary contacts of individuals such as family members, friends, school teachers, or college instructors who can act as gatekeepers to help suicidal people. The training is being given as an online course, too, and can be an efficient method to extend help to such individuals (50). These trained individuals will act as a bridge between people at suicide risk and mental health professionals who can help.

Older adults who are not very familiar with the use of technology-based interfaces for communication might experience social cut off and loneliness. Therefore, training the caregivers using short counseling videos on taking care of the elderly who are at risk and enabling them to tackle feelings of loneliness might be helpful. Brief online interventions for interpersonal therapy where the caregivers can be trained to address low levels of belongingness can be used (51). Techniques such as activity scheduling, cognitive exercises, and other behavior therapy techniques based on reinforcement, home-based tasks can be implemented by caregivers and family members so that older adults can engage themselves in constructive activities (52, 53). These activities can lessen feelings of burdensomeness and encourage productivity. Virtual social support via social media can compensate for feelings of isolation and challenges of belongingness (54).

Mindfulness-based therapies, especially mindfulness meditation techniques, mindfulness-based stress reduction strategies through videos and audios, and mindfulness-based cognitive therapy techniques, can address stress related to burnout, compassion fatigue, sleep problems, negative thoughts, and overwhelming emotions in healthcare professionals and frontline workers. Brief protocols of the above-mentioned techniques can be practiced in workplaces and hospitals as a routine practice. Several studies have demonstrated the efficacy of mindfulness-based cognitive therapy techniques in reducing depressive cognition and suicidal cognition (55–57). Brief cognitive therapy sessions through videoconferencing can be done, and the client can be assigned tasks and worksheets that they can return via email/ texts (58).

The COVID-19 pandemic is a crisis that is going to last for a while. As countries are struggling to deal with the consequences of the pandemic, the rise in psychiatric disorders and suicidal behaviors is alarming. However, with the right approaches, such as spreading awareness, strengthening telepsychotherapy, teleconsultations, and promoting self-help strategies, the frequency of suicide risk can be reduced.

## Author Contributions

SR, DG, and TS conceptualized the research topic. SR and DG prepared the initial draft. TS, SV, and YA reviewed the initial draft and suggested changes. SR, DG, TS, SV, and YA contributed to the preparation of the final manuscript. All authors contributed to the article and approved the submitted version.

## Conflict of Interest

The authors declare that the research was conducted in the absence of any commercial or financial relationships that could be construed as a potential conflict of interest.
